# A hierarchical fusion strategy of deep learning networks for detection and segmentation of hepatocellular carcinoma from computed tomography images

**DOI:** 10.1186/s40644-024-00686-8

**Published:** 2024-03-26

**Authors:** I-Cheng Lee, Yung-Ping Tsai, Yen-Cheng Lin, Ting-Chun Chen, Chia-Heng Yen, Nai-Chi Chiu, Hsuen-En Hwang, Chien-An Liu, Jia-Guan Huang, Rheun-Chuan Lee, Yee Chao, Shinn-Ying Ho, Yi-Hsiang Huang

**Affiliations:** 1 Division of Gastroenterology and Hepatology, Department of Medicine, Taipei Veterans General Hospital, Taipei, Taiwan; 2 School of Medicine, National Yang Ming Chiao Tung University, Taipei, Taiwan; 3 Institute of Bioinformatics and Systems Biology, National Yang Ming Chiao Tung University, Hsinchu, Taiwan; 4 Institute of Computer Science and Engineering, National Yang Ming Chiao Tung University, Hsinchu, Taiwan; 5 Department of Radiology, Taipei Veterans General Hospital, Taipei, Taiwan; 6 National Taiwan University School of Medicine, Taipei, Taiwan; 7 Cancer Center, Taipei Veterans General Hospital, Taipei, Taiwan; 8 Department of Biological Science and Technology, National Yang Ming Chiao Tung University, Hsinchu, Taiwan; 9 Center for Intelligent Drug Systems and Smart Bio-devices (IDS 2 B), National Yang Ming Chiao Tung University, Hsinchu, Taiwan; 10 College of Health Sciences, Kaohsiung Medical University, Kaohsiung, Taiwan; 11 Institute of Clinical Medicine, National Yang Ming Chiao Tung University, Taipei, Taiwan; 12 Healthcare and Service Center, Taipei Veterans General Hospital, Taipei, Taiwan

**Keywords:** Hepatocellular carcinoma, Deep learning, Segmentation, Detection, Computed tomography

## Abstract

**Background:**

Automatic segmentation of hepatocellular carcinoma (HCC) on computed tomography (CT) scans is in urgent need to assist diagnosis and radiomics analysis. The aim of this study is to develop a deep learning based network to detect HCC from dynamic CT images.

**Methods:**

Dynamic CT images of 595 patients with HCC were used. Tumors in dynamic CT images were labeled by radiologists. Patients were randomly divided into training, validation and test sets in a ratio of 5:2:3, respectively. We developed a hierarchical fusion strategy of deep learning networks (HFS-Net). Global dice, sensitivity, precision and F1-score were used to measure performance of the HFS-Net model.

**Results:**

The 2D DenseU-Net using dynamic CT images was more effective for segmenting small tumors, whereas the 2D U-Net using portal venous phase images was more effective for segmenting large tumors. The HFS-Net model performed better, compared with the single-strategy deep learning models in segmenting small and large tumors. In the test set, the HFS-Net model achieved good performance in identifying HCC on dynamic CT images with global dice of 82.8%. The overall sensitivity, precision and F1-score were 84.3%, 75.5% and 79.6% per slice, respectively, and 92.2%, 93.2% and 92.7% per patient, respectively. The sensitivity in tumors < 2 cm, 2–3, 3–5 cm and > 5 cm were 72.7%, 92.9%, 94.2% and 100% per patient, respectively.

**Conclusions:**

The HFS-Net model achieved good performance in the detection and segmentation of HCC from dynamic CT images, which may support radiologic diagnosis and facilitate automatic radiomics analysis.

**Supplementary Information:**

The online version contains supplementary material available at 10.1186/s40644-024-00686-8.

## Introduction

Hepatocellular carcinoma (HCC) is the sixth most common cancer and the fourth leading cause of cancer-related death in the world [1]. Dynamic computed tomography (CT) scan plays an important role in the diagnosis, staging and treatment decision of patients with HCC [2]. However, it requires professional and experienced radiologists to interpret hundreds slices of CT images per patient to detect HCC and determine the stage by measuring the size and number of HCC. After long working time, reading fatigue may lead to inefficiency of work, miss diagnosis, and even harm to physical and mental health [3]. Therefore, the artificial intelligence (AI)-assisted automatic detection and segmentation of HCC from dynamic CT images is in urgent need to assist radiologists. Besides, radiomics information from CT images may also have implication in predicting cancer outcomes [4]. Our recent study showed that by incorporating radiomic features in AI-derived prediction model could improve the prediction accuracy of HCC recurrence after resection [5]. The automatic segmentation of HCC may facilitate automatic radiomic analysis for AI-derived prediction models.

In the past, some studies have proposed automatic segmentation methods for liver tumors, but most of the studies used small datasets and only portal-venous phase CT images, which may not be sufficient to develop an accurate AI-detection model [6–13]. In clinical practice, radiologists read dynamic CT images including non-contrast, arterial, and portal-venous phase images slice-by-slice during interpretation. Due to the diverse heterogeneity of HCC, feature extraction methods and network architectures are important issues to such a difficult task. Model-based methods relied on handcrafted or non-handcrafted features of the image. With sufficient data and computational capability, deep learning may extract non-handcrafted features with better representation capability. Due to the powerful ability of deep learning, it could be easy to have overfitting by a single model. Using models of different learning strategies on a specific task in the method to collaborate might be a way to avoid the overfitting problem. The aim of this study is to develop a deep learning based network with multiple strategies for detection and segmentation of HCC from dynamic CT images.

## Materials and methods

### Patients

This study was approved by the Institutional Review Board, Taipei Veterans General Hospital, which complied with standards of the Declaration of Helsinki and current ethical guidelines. Due to the retrospective nature of the study, the Institutional Review Board waived the need for written informed consent. The identifying information of the enrolled subjects has been delinked and therefore authors could not access the information.

From October 1, 2007 to August 31, 2019, 884 consecutive HCC patients receiving surgical resection and radiofrequency ablation (RFA) in Taipei Veterans General Hospital with available CT images before treatment were retrospectively screened. The inclusion criteria were: (1) Age ≥ 20 years; (2) Available CT image within 3 months prior to surgical resection or RFA; (3) No other loco-regional treatment prior to surgical resection or RFA. Patients were excluded by the following criteria: (1) without complete dynamic contrast-enhanced CT (CECT) images including non-contrast phase, arterial phase and portal venous phase (*n* = 87); (2) poor image quality or unable to align the dynamic CT images according to the Z-axis (*n* = 202). Finally, 595 HCC patients with complete dynamic CECT images were enrolled in this study. HCC was diagnosed before surgery or RFA by CECT or magnetic resonance imaging (MRI), which fulfilled the diagnostic criteria of the American Association for the Study of Liver Diseases (AASLD) treatment guidelines for HCC [14] or was confirmed pathologically after surgery and RFA.

### CECT image segmentation

The image acquisition protocols of CT scanners involved in the present study are shown in Table S1. Interpretation and tumor segmentation of all CECT images of the 595 patients were performed by three radiologists who were blinded to the clinical and pathological data. The labeling of the HCC in dynamic CT images included the non-contrast phase, arterial phase and portal-venous phase. The three radiologists had read > 2,000 liver CT studies per year for at least 5 years. When contouring the tumor, the edge of the observed focal lesion within the liver was defined as an imaging appearance that is distinctive from the background according to the Liver Reporting and Data System (LI-RADS) [2, 15]. The contours of the liver were also labelled in 200 cases for training of segmentation of the liver. For evaluation of the HFS-Net model, ground truth tumor compartments were delineated manually. This was performed using a semiautomatic approach with subsequent manual editing (IntelliSpace Discovery; Philips Healthcare, Netherlands), performed by the three experienced radiologists [5].

### CT image processing

The characteristics of the CT image dataset were shown in Table 1. The original CT images were all 3D images stacked by 512 × 512 2D slices. Before the experiment, this study used downsampling to reduce all slices into a size of 256 × 256 and adjusted all CT image scale to 1 pixel equal to 1.4 mm. We aligned the dynamic CT images according to the Z-axis coordinates of the slices and superimposed them into a three-channel image for use. After alignment, 24,810 images (a total of 74,430 slices) were enrolled for analysis.

**Table 1:** Characteristics of the hepatocellular carcinoma

Dataset	Case number	Tumor size (cm)				Tumor number		BCLC stage		
		< 2	2–3	> 3–5	> 5	1	> 1	0	A	B
All	595	93	133	178	191	500	95	83	440	72
Training	298	42	78	81	97	245	53	38	220	40
Validation	118	18	27	45	28	104	14	14	95	9
Test	179	33	28	52	66	151	28	31	125	23

### Study design for HFS-Net

To design the best architecture of HFS-Net for HCC detection and segmentation, an ablation study was first conducted for evaluating various architectures of deep learning models. We randomly selected 491 cases from a total of 595 patients with a broad distribution of various tumor sizes was conducted for the ablation study.

The whole dataset was used to design and evaluate HFS-Net, which was randomly divided into non-overlapping training, validation, and test datasets in a 5:2:3 ratio. The training, validation, and test sets have 298, 118, and 179 patients, respectively. Each patient had a different number of slices and all were analyzed. The training set is used for constructing the HFS-Net model, while the validation set is used for tuning and optimization of model parameters. We then evaluate the performance of the HFS-Net model on a separate test set. HFS-Net was expected to combine the best sub-models by thorough evaluation of the candidate sub-models.

Therefore, the splitting strategy of the data and the construction of the model are independent in the two stages, and the purposes in the two stages are different. There is no comparison between the experimental results from the two stages, and the constructed models in the ablation experiments are not used in the final HFS-Net model. Instead, it uses the conclusions drawn from the ablation experiments as the background knowledge for model construction.

### Ablation study design for HFS-Net development

The ablation study investigates the performance of the proposed AI system HFS-Net by evaluating certain sub-models to understand the contribution and tolerance of the sub-models to the overall system. This study aims to discern the individual and combined strength of various sub-models in various scenarios. The influencing factors of system performance include architectures (U-Net, DenseU-net, Hyper-DenseU-Net, and R2U-Net), phases (non-contrast, arterial, portal-venous, and dynamic phases), loss functions (cross entropy, focal loss, median frequency balance, and dice loss), tumor sizes (the longest tumor axes in slices), assessment (dice score for model’s segmentation ability and tumor detection rate) and the fusion strategy.

The final stage of the HFS-Net design involves a comparison between 2D HFS-Net and the more comprehensive 3D HFS-Net. 2D HFS-Net leveraged the initial stages of hierarchical architecture and 2-D spatial features, while 3D HFS-Net incorporates a complete hierarchical architecture with fusion strategy and 3-D spatial features. This comparison helps to thoroughly evaluate the impact of hierarchical structures and fusion strategies to enhance the segmentation and detection of HCC.

### Neural network architecture of HFS-Net

**Fig. 1:**
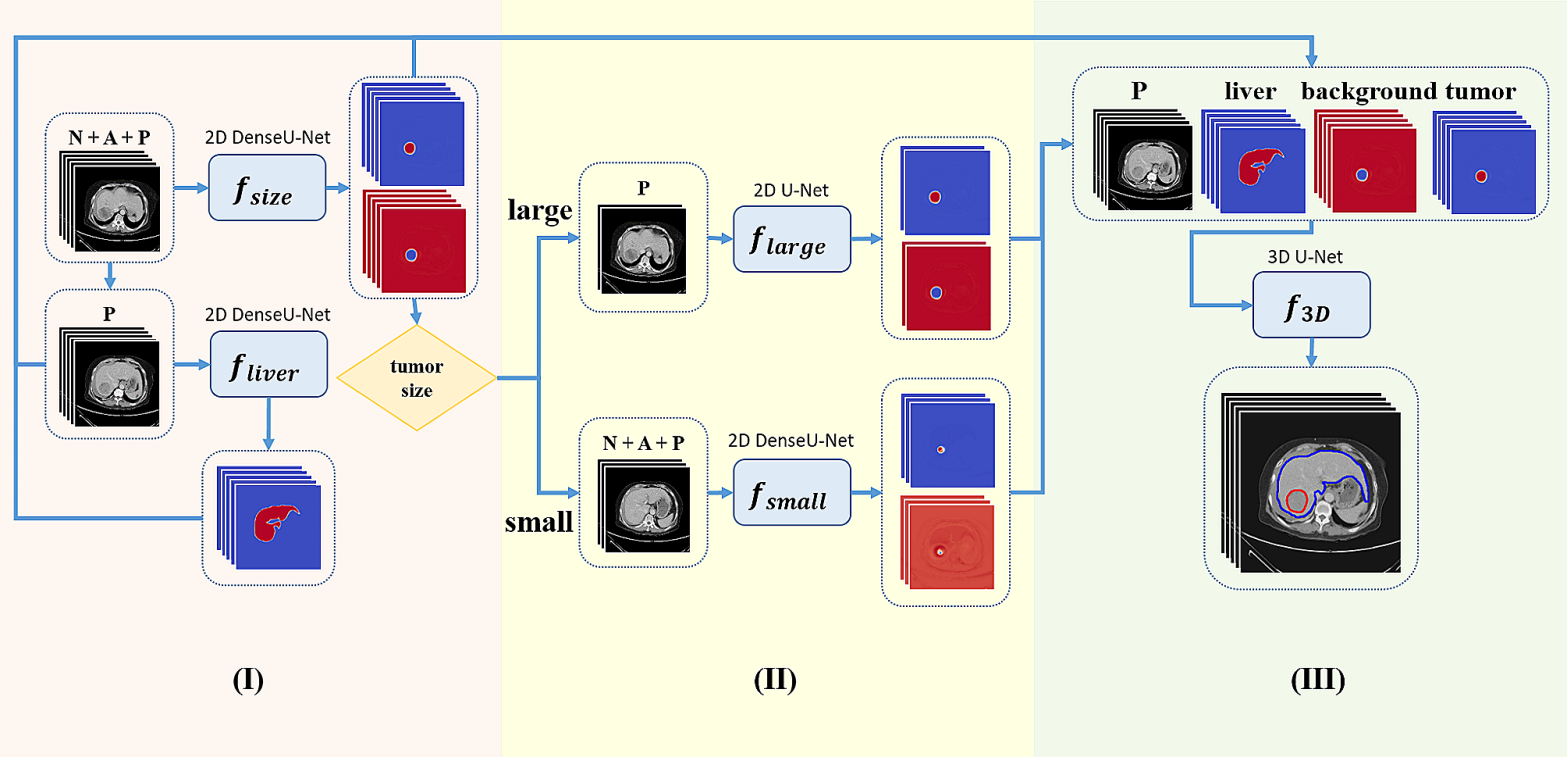
The HFS-Net data flow for liver and tumor segmentation. Stage I: Identify tumor’s longest axis in every slice of a case. Stage II: Accord tumor size by feeding the CT slice which the longest axis of tumors over 30 pixels in the slice for *f*_*large*_ computation and which the longest axis less than 30 pixels of tumors in the slice for *f*_*small*_ computation. Stage III: Combine venous phases of CT images and results of *f*_*liver*_, *f*_*size*_, *f*_*large*_, and *f*_*small*_ as input of *f*_*3D*_ computation for getting final segmentation of tumors

Figure 1 showed the data flow of our proposed HFS-Net method for liver and tumor detection and segmentation according to results of the ablation study. We cascaded five sub-models trained by different learning strategies (Table 2). HFS-Net consisted of three stages based on U-Net and DenseU-Net. The first stage of HFS-Net is the liver and tumor segmentation with tumor size estimation using 2D DenseU-Net, where every slice of the entire CT scan case was taken as input. The second stage is the divide-and-conquer stage. According to the tumor size calculated in the first stage, the tumors with the longest axis less than or equal to *m* pixels in the slice are assigned to the smaller tumor group, and those with more than *m* pixels are assigned to the larger tumor group. This study uses *m* = 30 pixels as the demarcation point, which is about 4.2 cm. In the divide-and-conquer stage, we use a customized model to adaptively segment large and small tumors in slices. For small-tumor groups, 2D DenseU-Net uses dynamic CT images to segment tumors, and for large-tumor groups, 2D U-Net uses portal-venous phase CT images to segment tumors. The third stage is the fusion strategy stage. This stage integrates the portal-venous phase CT image, the outcomes of the previous two stages and the segmentation of ​​the liver, and uses 3D U-Net to segment the final result of the 3D liver tumor. The detailed modeling and learning strategies of HFS-Net are described in Supplementary Methods.

**Table 2:** Function strategy in the HFS-Net

	Phase	Neural network	Loss function
*f*_*liver*_	Portal-venous	2D DenseU-Net	cross entropy + dice loss
*f*_*size*_	Dynamic	2D DenseU-Net	focal loss + dice loss
*f*_*large*_	Portal-venous	2D U-Net	cross entropy
*f*_*small*_	Dynamic	2D DenseU-Net	focal loss + dice loss
*f*_*3D*_	Portal-venous	3D U-Net	focal loss + dice loss

Dynamic CT images include non-contrast, arterial, and portal-venous phases

### Evaluation of the performance of the HFS-Net model

We used dice per case and dice global of Jaccard similarity as metrics to evaluate the performance of segmentation. Dice per case represents the average dice of the case, and dice global is the dice score that combines all the slices as a case to calculate. We used sensitivity, precision, and F1-score to evaluate detection performance. When evaluating the performance of detection, the criterion for successful detection is defined as the dice global that overlaps the model results with the corresponding tumor labels exceeding *θ*. Otherwise, it is a false positive. If the model fails to detect a tumor in a slice, it is considered a false negative. We set *θ* = 0.2 to require a significant, but not exact overlap as post study did [16]. In order to analyze the detection effect of the model more deeply, we designed four indicators to evaluate the detection performance of the model, including overall detection performance of tumors in slices (Per tumor volume), mean detection performance of tumors in slices (Per tumor cut), abnormal slice detection (Per slice) and abnormal case detection (Per patient).

The overall detection performance of tumors in slices means to use the tumors in all slices as the denominator to evaluate the performance of tumor detection. The mean detection performance of tumors in slices means to use the tumors in all slices of the case as the denominator to evaluate the performance of each case on average. Abnormal slice detection means the ability to detect at least one of the tumors in a slice with tumors, and abnormal case detection means the ability to detect at least one of the tumors in a case with tumors.

### Experiment environment

In this study, we used Nvidia GeForce RTX 2080 Ti (12GB) as the GPU, Intel(R) Xeon(R) Gold 6136 CPU @ 3.00 Ghz as the CPU, and used the CentOS Linux release 8.3.2011 as operating system. The method was implemented with python3.7 and Pytorch packages [17].

## Results

### HCC characteristics

The characteristics of the HCC were shown in Table 1. Among the 595 patients with HCC, 93 (15.6%), 133 (22.4%), 178 (29.9%) and 191 (32.1%) cases had HCC smaller than 2 cm, 2 to 3 cm, larger than 3 to 5 cm, and larger than 5 cm, respectively. Majority of the HCC were single tumor (84%) and in the BCLC stages 0 and A (13.9% and 73.9%, respectively).

### HFS-Net model development and fusion strategy approach

To evaluate the performance of the customized sub-models of HFS-Net in specific tumor size, we used 294 cases (37,608 slices) of CT images to train the model, 61 cases (7,833 slices) for validation, and 136 cases (12,576 slices) for test in the first part of the experiment. The longest axis of the tumor in the slice was used as the size basis. At first, we developed a 2D DenseU-Net model for liver segmentation, which may facilitate further HCC detection within the liver. The dice per case and dice global of the 2D DenseU-Net model for liver segmentation were 95.2% and 95.3%, respectively, in the test set (Table S2).

We applied U-Net and DenseU-Net to CT images of various phases to explore the phase effects on the deep learning models. The U-Net model using the portal-venous phase images performed best for tumors above 30 pixels, whereas the DenseU-Net model using dynamic CT images performed best for tumors with a size of 10 to 30 pixels (Table S3). The DenseU-Net model with dynamic CT images achieved the highest dice score of 48.9%. The second-highest performance was achieved by the DenseU-Net model using portal-venous phase images, with a dice score of 43.83%.

Then we used the same dataset to test the performance of models using different neural network architectures, including U-Net, DenseU-Net, R2U-Net, Hyper-DenseU-Net, and Single Dense Path U-Net. The latter two are designed for multimodal medical imaging [18, 19]. The results showed that the U-Net model using portal-venous phase images performed best for tumors above 30 pixels, achieving an average dice score of 87.56% in large tumor segmentation, whereas the DenseU-Net model using dynamic CT images performed best for tumors with a size of 10 to 30 pixels (Table S4).

In the next step, we tested the U-Net and DenseU-Net models with different loss functions, including cross-entropy, focal loss, median frequency balance focal loss, and dice loss. The results showed that the DenseU-Net model using dynamic CT images with focal loss plus dice loss as the loss function could improve the segmentation performance for smaller tumors, while the U-Net model using portal-venous phase images with cross-entropy performed best for larger tumors (Table S5).

Finally, in order to test whether the hierarchical fusion strategy of HFS-Net is effective, we evaluated the test performance of 2D HFS-Net and 3D HFS-Net (Table S6). The 2D HFS-Net means that HFS-Net contains hierarchical but does not contain fusion strategy, whereas the 3D HFS-Net used both hierarchical and fusion strategies. The results showed that the 2D HFS-Net model could improve the segmentation performance and detection rate of tumors with a size of 10 to 50 pixels after combining U-Net and DenseU-Net models. Since the 3D HFS-Net model considered the features of the 3D space and integrated the outcomes of models of different strategies, the 3D HFS-Net model not only improved the segmentation performance and detection rate as compared to the 2D HFS-Net model, but also greatly reduced the false detection rate. The data flow of the finally proposed HFS-Net model for liver and tumor detection and segmentation was shown in Fig. 1.

### Performance of the HFS-Net model

In this section, we randomly divide the complete dataset in a ratio of 5:2:3, used 298 cases (37,824 slices) of CT images to train the model, 118 cases (15,423 slices) for validation, and 179 cases (22,803 slices) for test. The training set fits the model, while the validation set tunes and optimizes the model. The final HFS-Net model was developed from the training and validation sets. We subsequently evaluate the performance of the model on a separate test set. The performance of the HFS-Net model for HCC detection and segmentation in the training, validation and test sets was shown in Table S7.

**Fig. 2:**
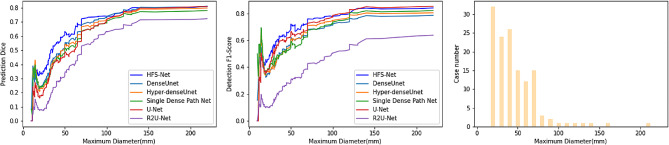
Performance of HFS-Net segmentation (left), detection (middle) and distribution of sizes of HCC (right)

We compared the HFS-Net model with other single-strategy models by using the test set. As shown in Fig. 2, the HFS-Net model generally had the highest prediction dice and F1-score compared to other single-strategy models, suggesting that the HFS-Net model outperforms other single-strategy models for the detection and segmentation of HCC from small to large tumor size.

**Fig. 3:**
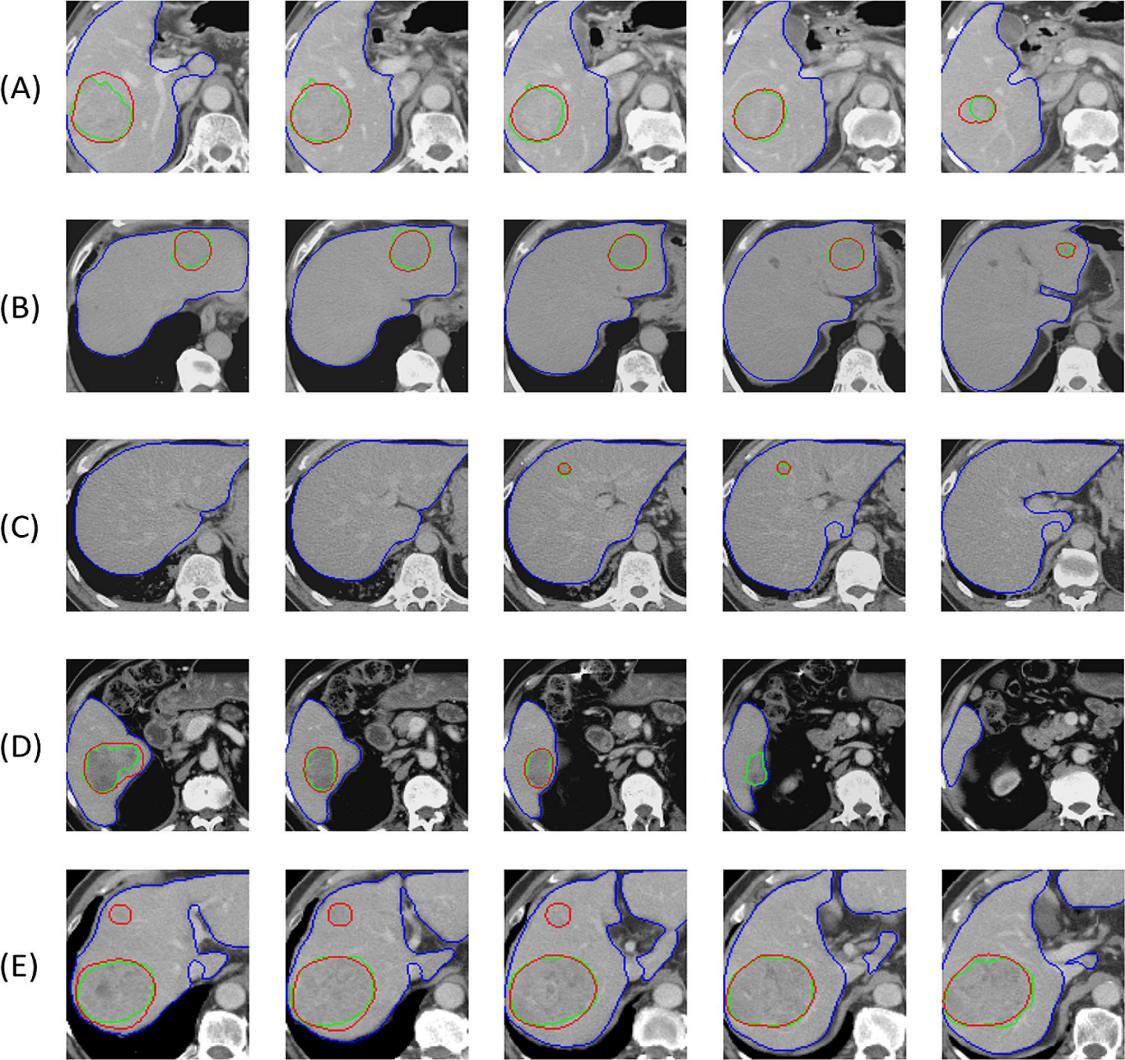
Examples of HFS-Net segmentation results from test dataset. **A** A 8.5 cm HCC representing large HCC (dice per case 88.1%). **B** A 4.6 cm HCC representing medium sized HCC (dice per case 80%). **A** A 8.5 cm HCC representing large HCC (dice per case 88.1%). **C** A 1.7 cm HCC representing BCLC stage 0 HCC (dice per case 83.6%). **D** A tumor was detected in the first three slices but missed in the marginal slice of the tumor. **E** A large tumor accurately segmented by HFS-Net, and a small tumor missed by radiologist labelling but detected by HFS-Net. The green line represents the radiologist’s label, the red line represents the output of HFS-Net for tumor margin, and the blue line represents the output of HFS-Net for liver margin

The performance of the HFS-Net model in the test set was shown in Table 3. The overall dice per case was 58.7%, and the dice global was 82.8%. The overall sensitivity, precision and F1-score were 84.3%, 75.5% and 79.6% per slice, respectively, and were 92.2%, 93.2% and 92.7% per patient, respectively. Notably, in analysis per patient, there is no negative case in the prediction. Therefore, the representativeness of the F1-score would be similar to that of the sensitivity. The sensitivity, precision and F1-score per patient of the HFS-Net model were generally higher than 90% for tumors larger than 2 cm. For tumors less than 2 cm, the accuracy of the HFS-Net model decreases but the sensitivity and precision per patient remained higher than 70%. Figure 3 showed the examples of HCC segmentation by the HFS-Net model in the test set. Our model could detect majority of the tumors with various sizes, although there were some differences in the tumor margin between the labelling by radiologists and the HFS-Net model (Fig. 3A-C). Some tumors could be detected in the slices of maximum tumor size but might be missed in the marginal slice of the tumor (Fig. 3D). The HFS-Net model could also detect few small tumors in individual CT slices which were initially missed by the radiologist’s labeling (Fig. 3E), and the labeling of these missed tumors could be corrected.

**Table 3:** Performance of HFS-Net in the test set

		Segmentation		Per tumor volume	Per tumor cut	Per slice	Per patient
All (*n* = 179)	Dice per case	58.7%	Sensitivity	88.9%	82.1%	84.3%	92.2%
	Dice global	82.8%	Precision	51.5%	67.6%	75.5%	93.2%
	MTD MAE	0.73 cm	F1-Score	65.3%	74.2%	79.6%	92.7%
1 ~ 2 cm (*n* = 33)	Dice per case	32.0%	Sensitivity	69.4%	52.4%	57.0%	72.7%
	Dice global	32.7%	Precision	45.5%	34.1%	39.5%	77.4%
	MTD MAE	0.56 cm	F1-Score	54.9%	41.3%	46.7%	75.0%
2 ~ 3 cm (*n* = 28)	Dice per case	47.3%	Sensitivity	90.3%	75.0%	77.8%	92.9%
	Dice global	55.5%	Precision	57.1%	58.7%	66.7%	92.9%
	MTD MAE	0.57 cm	F1-Score	70.0%	65.9%	71.7%	92.9%
3 ~ 5 cm (*n* = 52)	Dice per case	60.4%	Sensitivity	89.1%	75.7%	79.8%	94.2%
	Dice global	69.1%	Precision	49.0%	60.8%	69.0%	94.2%
	MTD MAE	0.48 cm	F1-Score	63.2%	67.4%	74.0%	94.2%
> 5 cm (*n* = 66)	Dice per case	76.1%	Sensitivity	98.5%	88.1%	89.5%	100%
	Dice global	84.5%	Precision	54.0%	75.6%	84.2%	100%
	MTD MAE	1.04 cm	F1-Score	65.7%	81.4%	86.8%	100%
1 tumor (*n* = 151)	Dice per case	58.2%	Sensitivity	89.2%	81.7%	83.7%	92.7%
	Dice global	84.2%	Precision	52.0%	66.7%	74.7%	93.3%
	MTD MAE	0.70 cm	F1-Score	65.7%	73.4%	78.9%	93.0%
> 1 tumors (*n* = 28)	Dice per case	62.9%	Sensitivity	87.5%	83.3%	86.0%	89.3%
	Dice global	78.1%	Precision	49.1%	70.6%	78.2%	92.6%
	MTD MAE	0.90 cm	F1-Score	62.9%	76.4%	82.0%	90.9%

MTD, maximum tumor diameter; MAE, mean absolute error

### Calculating time

HFS-Net inference was mainly divided into three stages: (1) reading model parameters and moving to GPU memory; (2) reading CT images to be tested; (3) the model inference. It took about 1.527 s to read the parameters trained by HFS-Net to the CPU, and about 3.453 s to copy HFS-Net from the CPU to the GPU. It took about 0.028 s to read the CT image to be tested, convert it into an array from dicom format and perform pre-processing for each slice. It took about 0.030 s to copy the array of the image to be tested to the GPU and perform inference for each slice. The step of reading model parameters only needs to be read once during the initial state, and multiple CT image inferences can be performed with once reading. Across the entire dataset, the average time for reading data to complete inference was 4.517 s, with a standard deviation of 0.877 s. By testing a case with 126 slices, the real execution time was 4.29 s. The demonstration of the HFS-Net model is shown in the supplementary movie. From the data presented, it is clear that the HFS-Net can complete its processing within 10 s.

## Discussion

In this study, we proposed a hierarchical fusion strategy of deep learning network called HFS-Net, which used a coarse-to-fine mechanism to automatically detect HCC from CT images. We found that dynamic CT images combined with deep learning methods could perform better than a single-phase image model in detecting and segmenting small tumors. We used the hierarchical fusion strategy to solve the single-model trade-off between small and large tumor segmentation of CT slices. Compared with the single-strategy model, our method has improved detection and segmentation performance in tumors of various sizes. Our method achieves acceptable performance for detecting and segmenting HCC and can interpret a patient’s CT scans within 10 s.

This study explored the effect of the dynamic CT model and proposed a novel deep learning architecture to segment liver and HCC in CT scans. HCC may show different brightness in different phases of CT images. Interpreting CT images of non-contrast, arterial and portal-venous phases is necessary for the clinical judgment of liver tumors. Although deep learning methods have achieved good results in the field of tumor segmentation in the past, they lacked consideration of different phase features of CT images [6–13]. In this study, we found that using dynamic CT images with DenseU-Net could improve the performance in segmenting smaller tumors. DenseU-Net’s dense block uses tightly connected paths to achieve an effective dynamic CT feature extraction method. A previous study indicated that DenseU-Net combined with focal loss is quite effective as a model architecture for small target segmentation [20].

U-Net is a popular method for image segmentation and has achieved success in many medical image segmentation tasks [21–23]. However, U-Net, which has less interaction of high-level features and low-level features, could not effectively learn the features of dynamic CT images. We found that U-Net using single portal-venous phase CT images to segment larger HCC had better performance. HCC in the portal-venous phase had clearer edges and were less likely to be confused by noise when segmenting larger tumors. In order to maximize the performance for both large and small tumors, we proposed a novel hierarchical fusion strategy of deep learning networks, which uses multi-task learning to customize suitable learning strategies for specific tasks and merge these strategies at the end to output more reliable results. Our results showed that the model segmentation and detection performance in each tumor size is better than other models using a single learning strategy.

The HFS-Net model could achieve a good performance in detecting HCC, and the sensitivity, precision and F1-score per patient were generally higher than 92%. When stratified by tumor size, the accuracy for detecting HCC larger than 2 cm remained higher than 92%, whereas the accuracy of the HFS-Net model decreases for tumors less than 2 cm, with the sensitivity and precision per patient for HCC less than 2 cm of 72.7% and 77.4%, respectively. Very early HCC may have less obvious enhancing pattern and may be difficult to be diagnosed by CT scan [24]. In clinical practice, some of the atypical HCC presented in CT scan were alternatively diagnosed by MRI or confirmed pathologically after resection or RFA [25]. More case numbers and additional image modalities might be needed to further improve the detection accuracy for small HCC. The imperfect detection rates of the HFS-Net model, especially for HCC less than 2 cm, indicate that the current AI-derived model could assist but not replace the interpretation of the radiologists.

The dice global of the HFS-Net model was 82.8%, indicating an acceptable accuracy of tumor segmentation. An accurate tumor segmentation may assist automatic measurement of tumor size and volume, which are important clinical parameters for staging, treatment decision and outcomes prediction [26, 27]. Moreover, automatic segmentation may facilitate radiomics analysis of HCC. Our recent study showed that the radiomics of HCC may contain significant prognostic information and radiomics analysis is increasingly adopted by AI-derived prognostic models for cancer outcomes [4, 5]. However, manual labeling of the liver and HCC were quite exhausting and time-consuming. When establishing the HCC labeling dataset in this study, it took about 1 min to interpret liver CT for detection of HCC and 10 min for labeling the tumor in multiple slices by a radiologist. After development of the HFS-Net model, it took only about 8 s for automatic detection and segmentation of HCC for one patient.

This study has some limitations. First, this is a retrospective study from a single center. The accuracy of the HFS-Net model need further external validation in the future. Second, this study only analyzed CT images with HCC. The detection ability for other benign and malignant liver tumors was not evaluated in this study. Clinical image datasets of different liver tumors to train the AI model for detection and differential diagnosis of other types of liver tumors are needed in the future. Furthermore, while the handling of breathing artifacts is an issue, our methodology did not explicitly focus on correcting or compensating for these artifacts. Therefore, incorporating image registration into the enhancement of the image processing methods may advance model performance. Third, although the dice global of the HFS-Net model achieved 82.8%, the accuracy was relatively lower for tumors with smaller size. Further research is needed to improve prediction accuracy for smaller tumors. Fourth, this study only included CT images from patients with earlier stage HCC prior to resection or RFA. Therefore, the current model could only detect intrahepatic HCC before treatment. Future works are needed to analyzed post-treatment CT images as well as extrahepatic lesions and vascular invasion.

In conclusion, by establishing a large dataset of CT images with HCC labeling, we developed a novel HFS-Net model, which performed better than other single-strategy models for both small and large HCC. The HFS-Net model could achieve a good performance in automatic detection and segmentation of HCC from dynamic CT images in only a few seconds. This model may support radiologic diagnosis and facilitate automatic radiomics analysis in the future.

### Electronic supplementary material

Below is the link to the electronic supplementary material.

Supplementary Material 1

Supplementary Material 2

## Data Availability

The data that support the findings of this study are available from the corresponding author upon reasonable request.

## Abbreviations

AASLD: American Association for the Study of Liver Diseases
AI: artificial intelligence
CT: computed tomography
CECT: contrast-enhanced computed tomography
HCC: hepatocellular carcinoma
HFS-Net: hierarchical fusion strategy of deep learning networks
RFA: radiofrequency ablation
