# Supplementary material for: A hierarchical fusion strategy of deep learning networks for detection and segmentation of hepatocellular carcinoma from computed tomography images
Source: Cancer Imaging. 2024 Mar 26;24:43. doi: 10.1186/s40644-024-00686-8 (PMC10964581; doi:10.1186/s40644-024-00686-8)
Supplement: Supplementary file 2 — Supplementary Material 2 [file 40644_2024_686_MOESM2_ESM.docx]

**SUPPLEMENTARY METHODS**

**Neural network architecture of HFS-Net**

Figure 2 shows the data flow of our proposed method for liver and tumor segmentation. We cascaded five sub-models trained by different learning strategies.

Let $I$ denote the dynamic images and $I_{p}$the portal-venous phase images. At stage (I), tumor size stage, the images were fed into $f_{liver}$ and $f_{size}$ to get liver segmentation and tumor segmentation probability map that denote as $X_{liver}$ and $X_{size}$ as follows.

(1)

$I\in R^{16n\times3\times256\times256}$, $\boldsymbol{I}_{\boldsymbol{p}}\in R^{16n\times1\times256\times256}$

$\boldsymbol{X}_{\boldsymbol{liver}}=f_{liver}\left( \boldsymbol{I}_{\mathbf{p}} \right)$, $\boldsymbol{X}_{\boldsymbol{liver}}\in R^{16n\times1\times256\times256}$

$\boldsymbol{X}_{\boldsymbol{size}}=f_{size}\left( \boldsymbol{I} \right)$, $\boldsymbol{X}_{\boldsymbol{size}}\in R^{16n\times2\times256\times256}$

After getting $X_{size}$, we calculate the longest axis of tumor in each slice and slices of a case would be assigned into small ($I_{small}$) or large ($I_{large}$) groups according to tumor size less or greater than m pixels at stage (II), divide-and-conquer stage. The smaller tumor group uses dynamic CT images for 2D DenseU-Net to get *X*_small_, while the larger tumor group uses portal-venous phase CT images for 2D U-Net to get $X_{large}$.

(2)

$X_{large}=f_{large}\left( I_{\mathrm{large}} \right)$, $X_{large}\in R^{16n\times2\times256\times256}$

$X_{small}=f_{small}\left( I_{small} \right)$, $X_{small}\in R^{16n\times2\times256\times256}$

At stage (III), fusion strategy stage, portal-venous phase CT images are combined with $X_{liver}$, $X_{large}$ and $X_{small}$. If any slice has larger positive area prediction in $X_{size}$ than in $X_{large}$ or $X_{small}$, we change the prediction of $X_{large}$ or $X_{small}$ to prediction of $X_{size}$ to maximize sensitivity. It is very effective to reduce false positives when we consider the 3D features at this stage. After combination, we fed the images array of cases into 3D-Unet in groups of 16. The 3D convolution layers learns the features from the different models based on a different strategy and outputs the final liver tumor segmentation result ($Y$).

(3)

$$Y=f_{3d}\left( I_{p} ,X_{liver},X_{size},X_{large},X_{small} \right)$$

**Modeling and learning strategy**

HFS-Net consists of five different task models, $f_{liver}$, $f_{size}$, $f_{large},f_{small}$ and $f_{3D}$, to segment liver tumors from CT volume. To have better performance in specific tasks, each model has different learning strategies. These strategies include the use of dynamic or portal-venous phase image features, the model architecture used, and the loss function. The strategy of each function has shown in Table 3. Each function is trained based on the tumor size that the task is dealing with. The method of specifically segmenting large tumors only used the data of large tumors in the training set to learn, the method of specifically segmenting small tumors only used the data of small tumors in the training set to learn, and the rest used the data of all training sets to learn. Here, m=30 pixels are used as the dividing point between large and small tumors, and the m value is considered from the perspective of the model, and has nothing to do with the clinical way to distinguish large and small tumors.

Portal-venous phase CT images have more obvious tumor edges, but they may have the noise in the liver. Therefore, it is necessary to refer to the dynamic CT image comparison during diagnosis. For liver and larger tumors, noise is less likely to be confused with them. Therefore, $f_{liver}$ and $f_{large}$ are suitable for using portal-venous phase CT images as input. For $f_{size}$ and $f_{small}$, it is necessary to consider a dynamic CT to avoid noise and tumor confusion. Although $f_{3D}$ use portal-venous phase CT image as input, it use features extracted from other models that use dynamic CT image features. The purpose of adding portal-venous phase CT image is to combine the elementary features of the image with the advanced deep learning extraction features to enhance the representation capability.

U-Net is widely used and promoted in medical imaging and has achieved good results. This study uses 2D U-Net and 3D U-Net modified from Olaf et al. and Kayalıbay et al. in $f_{large}$ and $f_{3D}$ as network structures, respectively.^1-2^ The densely connected structure in the DenseU-Net can concatenate low-level features and high-level features in a block. It can effectively achieve feature reuse and alleviate the problem of gradient vanishment.^3^ Dong et al. indicated that this structure was beneficial for the segmentation task of small targets, so this research used this architecture on $f_{liver}$, $f_{size}$, and $f_{small}$.^4^

The loss function is an indicator that evaluates the different between the model distribution and data distribution. The model is trained by adjusting model parameters through gradient descent to reduce loss. Cross-entropy is the most common and general loss function, but its disadvantage is that it only considers the overall model performance, so difficult or rare problems are easily overlooked.^5^ To solve this problem, focal loss adds weight to the difficult problems, and dice loss considers the overlap ratio of the segmentation results rather than the absolute number to increase the weight of rare data types.^6-7^ Mixing dice loss and other loss function methods can make the training process of the model more stable.^8^ CT images usually contain a large number of background pixels and a few tumor pixels in the image, and small tumors has been known as difficult problem in the task but an important problem in the auxiliary diagnosis. So we used focal loss plus dice loss is used as the loss function of $f_{size}$, $f_{small}$, and $f_{3D}$, while large tumors use cross entropy as the loss function.

**Method detail**

We provide the code implementations for the U-Net and DenseU-Net architectures used in the HFS-Net sub-models in the supplementary files. These architectures are integral to HFS-Net's ability to segment liver and tumor structures effectively in medical imaging. For instance, the $f_{size}$, and $f_{small}$ utilize DenseU-Net architecture with triple-phase images, while the $f_{large}$ employs the U-Net architecture with portal-venous phase images.

All models were trained using the Adam optimizer, with an initial learning rate 0.0001. During training, if the loss on the training set did not decrease throughout 20 epochs, the learning rate was reduced to 90% of its current value. During training, each batch randomly employed data augmentation techniques such as flipping, cropping, and dropout to mitigate the risk of overfitting. These methods ensure a more robust model by enhancing the variability and complexity of the training data.

U-Net source code:

**import** torch

**import** torch**.**nn **as** nn

**class** **conv_block(**nn**.**Module**):**

**def** __init__**(**self**,**ch_in**,**ch_out**):**

**super(**conv_block**,**self**).**__init__**()**

self**.**conv **=** nn**.**Sequential**(**

nn**.**Conv2d**(**ch_in**,** ch_out**,** kernel_size**=**3**,**stride**=**1**,**padding**=**1**,**bias**=True),**

nn**.**BatchNorm2d**(**ch_out**),**

nn**.**ReLU**(**inplace**=True),**

nn**.**Conv2d**(**ch_out**,** ch_out**,** kernel_size**=**3**,**stride**=**1**,**padding**=**1**,**bias**=True),**

nn**.**BatchNorm2d**(**ch_out**),**

nn**.**ReLU**(**inplace**=True)**

**)**

**def** forward**(**self**,**x**):**

x **=** self**.**conv**(**x**)**

**return** x

**class** **up_conv(**nn**.**Module**):**

**def** __init__**(**self**,**ch_in**,**ch_out**):**

**super(**up_conv**,**self**).**__init__**()**

self**.**up **=** nn**.**Sequential**(**

nn**.**Upsample**(**scale_factor**=**2**),**

nn**.**Conv2d**(**ch_in**,**ch_out**,**kernel_size**=**3**,**stride**=**1**,**padding**=**1**,**bias**=True),**

nn**.**BatchNorm2d**(**ch_out**),**

nn**.**ReLU**(**inplace**=True)**

**)**

**def** forward**(**self**,**x**):**

x **=** self**.**up**(**x**)**

**return** x

**class** **U_Net(**nn**.**Module**):**

**def** __init__**(**self**,**img_ch**=**3**,**output_ch**=**1**):**

**super(**U_Net**,**self**).**__init__**()**

self**.**Maxpool **=** nn**.**MaxPool2d**(**kernel_size**=**2**,**stride**=**2**)**

self**.**Conv1 **=** conv_block**(**ch_in**=**img_ch**,**ch_out**=**64**)**

self**.**Conv2 **=** conv_block**(**ch_in**=**64**,**ch_out**=**128**)**

self**.**Conv3 **=** conv_block**(**ch_in**=**128**,**ch_out**=**256**)**

self**.**Conv4 **=** conv_block**(**ch_in**=**256**,**ch_out**=**512**)**

self**.**Conv5 **=** conv_block**(**ch_in**=**512**,**ch_out**=**1024**)**

self**.**Up5 **=** up_conv**(**ch_in**=**1024**,**ch_out**=**512**)**

self**.**Up_conv5 **=** conv_block**(**ch_in**=**1024**,** ch_out**=**512**)**

self**.**Up4 **=** up_conv**(**ch_in**=**512**,**ch_out**=**256**)**

self**.**Up_conv4 **=** conv_block**(**ch_in**=**512**,** ch_out**=**256**)**

self**.**Up3 **=** up_conv**(**ch_in**=**256**,**ch_out**=**128**)**

self**.**Up_conv3 **=** conv_block**(**ch_in**=**256**,** ch_out**=**128**)**

self**.**Up2 **=** up_conv**(**ch_in**=**128**,**ch_out**=**64**)**

self**.**Up_conv2 **=** conv_block**(**ch_in**=**128**,** ch_out**=**64**)**

self**.**Conv_1x1 **=** nn**.**Conv2d**(**64**,**output_ch**,**kernel_size**=**1**,**stride**=**1**,**padding**=**0**)**

**def** forward**(**self**,**x**):**

# encoding path

x1 **=** self**.**Conv1**(**x**)**

x2 **=** self**.**Maxpool**(**x1**)**

x2 **=** self**.**Conv2**(**x2**)**

x3 **=** self**.**Maxpool**(**x2**)**

x3 **=** self**.**Conv3**(**x3**)**

x4 **=** self**.**Maxpool**(**x3**)**

x4 **=** self**.**Conv4**(**x4**)**

x5 **=** self**.**Maxpool**(**x4**)**

x5 **=** self**.**Conv5**(**x5**)**

# decoding + concat path

d5 **=** self**.**Up5**(**x5**)**

d5 **=** torch**.**cat**((**x4**,**d5**),**dim**=**1**)**

d5 **=** self**.**Up_conv5**(**d5**)**

d4 **=** self**.**Up4**(**d5**)**

d4 **=** torch**.**cat**((**x3**,**d4**),**dim**=**1**)**

d4 **=** self**.**Up_conv4**(**d4**)**

d3 **=** self**.**Up3**(**d4**)**

d3 **=** torch**.**cat**((**x2**,**d3**),**dim**=**1**)**

d3 **=** self**.**Up_conv3**(**d3**)**

d2 **=** self**.**Up2**(**d3**)**

d2 **=** torch**.**cat**((**x1**,**d2**),**dim**=**1**)**

d2 **=** self**.**Up_conv2**(**d2**)**

d1 **=** self**.**Conv_1x1**(**d2**)**

**return** torch**.**sigmoid**(**d1**)**

DenseU-Net source code:

**import** torch

**import** torch**.**nn **as** nn

**class** **Dense_Block(**nn**.**Module**):**

**def** __init__**(**self**,** in_channels**,** out_channels**):**

**super(**Dense_Block**,** self**).**__init__**()**

self**.**conv1 **=** nn**.**Conv2d**(**in_channels **=** in_channels**,** out_channels **=** out_channels**,** kernel_size **=** 3**,** stride **=** 1**,** padding **=** 1**)**

self**.**bn1 **=** nn**.**BatchNorm2d**(**out_channels**)**

self**.**relu **=** nn**.**ReLU**(**inplace **=** **True)**

self**.**conv2 **=** nn**.**Conv2d**(**in_channels **=** out_channels**+**in_channels**,** out_channels **=** out_channels**,** kernel_size **=** 3**,** stride **=** 1**,** padding **=** 1**)**

self**.**bn2 **=** nn**.**BatchNorm2d**(**out_channels**)**

self**.**conv3 **=** nn**.**Conv2d**(**in_channels **=** 2*****out_channels**+**in_channels**,** out_channels **=** out_channels**,** kernel_size **=** 1**,** stride **=** 1**,** padding **=** 0**)**

self**.**bn3 **=** nn**.**BatchNorm2d**(**out_channels**)**

**def** forward**(**self**,** x**):**

conv1 **=** self**.**relu**(**self**.**bn1**(**self**.**conv1**(**x**)))** # h * w * 64

conv2 **=** self**.**relu**(**self**.**bn2**(**self**.**conv2**(**torch**.**cat**([**x**,** conv1**],** dim**=**1**))))** # h * w * 64

conv3 **=** self**.**relu**(**self**.**bn3**(**self**.**conv3**(**torch**.**cat**([**x**,** conv1**,** conv2**],** dim**=**1**))))** # h * w * 64

**return** conv3

**class** **Up_Block(**nn**.**Module**):**

**def** __init__**(**self**,** in_channels**,** out_channels**):**

**super(**Up_Block**,** self**).**__init__**()**

self**.**TransposeConv **=** nn**.**ConvTranspose2d**(**in_channels**=**in_channels**,**out_channels**=**out_channels**,**kernel_size**=**2**,**stride**=**2**)**

self**.**bn1 **=** nn**.**BatchNorm2d**(**out_channels**)**

self**.**relu **=** nn**.**ReLU**(**inplace **=** **True)**

**def** forward**(**self**,** x**):**

x_up **=** self**.**relu**(**self**.**bn1**(**self**.**TransposeConv**(**x**)))**

**return** x_up

**class** **Up_Dense_Block(**nn**.**Module**):**

**def** __init__**(**self**,** in_channels**,** out_channels**):**

**super(**Up_Dense_Block**,** self**).**__init__**()**

self**.**conv0 **=** nn**.**Conv2d**(**in_channels**=**in_channels**,** out_channels**=**out_channels**,** kernel_size**=**1**,** stride**=**1**,** padding**=**0**)**

self**.**bn0 **=** nn**.**BatchNorm2d**(**out_channels**)**

self**.**relu **=** nn**.**ReLU**(**inplace **=** **True)**

self**.**conv1 **=** nn**.**Conv2d**(**in_channels **=** out_channels**,** out_channels **=** out_channels**,** kernel_size **=** 3**,** stride **=** 1**,** padding **=** 1**)**

self**.**bn1 **=** nn**.**BatchNorm2d**(**out_channels**)**

self**.**conv2 **=** nn**.**Conv2d**(**in_channels **=** out_channels*****2**,** out_channels **=** out_channels**,** kernel_size **=** 3**,** stride **=** 1**,** padding **=** 1**)**

self**.**bn2 **=** nn**.**BatchNorm2d**(**out_channels**)**

self**.**conv3 **=** nn**.**Conv2d**(**in_channels **=** out_channels*****3**,** out_channels **=** out_channels**,** kernel_size **=** 1**,** stride **=** 1**,** padding **=** 0**)**

self**.**bn3 **=** nn**.**BatchNorm2d**(**out_channels**)**

**def** forward**(**self**,** x**):**

x **=** self**.**relu**(**self**.**bn0**(**self**.**conv0**(**x**)))**

conv1 **=** self**.**relu**(**self**.**bn1**(**self**.**conv1**(**x**)))** # h * w * 64

conv2 **=** self**.**relu**(**self**.**bn2**(**self**.**conv2**(**torch**.**cat**([**x**,** conv1**],** dim**=**1**))))** # h * w * 64

conv3 **=** self**.**relu**(**self**.**bn3**(**self**.**conv3**(**torch**.**cat**([**x**,** conv1**,** conv2**],** dim**=**1**))))** # h * w * 64

**return** conv3

**class** **DenseU_Net(**nn**.**Module**):**

**def** __init__**(**self**,**img_ch**=**3**,**output_ch**=**1**):**

**super(**DenseU_Net**,**self**).**__init__**()**

self**.**Maxpool **=** nn**.**MaxPool2d**(**kernel_size**=**2**,**stride**=**2**)**

self**.**Conv1 **=** Dense_Block**(**in_channels**=**img_ch**,**out_channels**=**64**)**

self**.**Conv2 **=** Dense_Block**(**in_channels**=**64**,**out_channels**=**128**)**

self**.**Conv3 **=** Dense_Block**(**in_channels**=**128**,**out_channels**=**256**)**

self**.**Conv4 **=** Dense_Block**(**in_channels**=**256**,**out_channels**=**512**)**

self**.**Conv5 **=** Dense_Block**(**in_channels**=**512**,**out_channels**=**512**)**

self**.**Up5 **=** Up_Block**(**in_channels**=**512**,**out_channels**=**512**)**

self**.**Up_conv5 **=** Up_Dense_Block**(**in_channels**=**1024**,** out_channels**=**512**)**

self**.**Up4 **=** Up_Block**(**in_channels**=**512**,**out_channels**=**512**)**

self**.**Up_conv4 **=** Up_Dense_Block**(**in_channels**=**1024**,** out_channels**=**256**)**

self**.**Up3 **=** Up_Block**(**in_channels**=**256**,**out_channels**=**256**)**

self**.**Up_conv3 **=** Up_Dense_Block**(**in_channels**=**512**,** out_channels**=**128**)**

self**.**Up2 **=** Up_Block**(**in_channels**=**128**,**out_channels**=**128**)**

self**.**Up_conv2 **=** Up_Dense_Block**(**in_channels**=**256**,** out_channels**=**64**)**

self**.**Up1 **=** Up_Block**(**in_channels**=**64**,**out_channels**=**64**)**

self**.**Up_conv1 **=** Up_Dense_Block**(**in_channels**=**128**,** out_channels**=**64**)**

self**.**Outconv **=** nn**.**Conv2d**(**64**,**output_ch**,**kernel_size**=**3**,**stride**=**1**,**padding**=**1**)**

**def** forward**(**self**,**x**):**

# encoding path

# x: 3*256*256

x1 **=** self**.**Conv1**(**x**)** # 64*256*256

x2 **=** self**.**Maxpool**(**x1**)** # 64*128*128

x2 **=** self**.**Conv2**(**x2**)** # 128*128*128

x3 **=** self**.**Maxpool**(**x2**)** # 128*64*64

x3 **=** self**.**Conv3**(**x3**)** # 256*64*64

x4 **=** self**.**Maxpool**(**x3**)** # 256*32*32

x4 **=** self**.**Conv4**(**x4**)** # 512*32*32

x5 **=** self**.**Maxpool**(**x4**)** # 512*16*16

x5 **=** self**.**Conv5**(**x5**)** # 512*16*16

x6 **=** self**.**Maxpool**(**x5**)** # 512*8*8

# decoding + concat path

d5 **=** self**.**Up5**(**x6**)** # 512*16*16

d5 **=** torch**.**cat**((**x5**,**d5**),**dim**=**1**)** # 1024*16*16

d5 **=** self**.**Up_conv5**(**d5**)** # 512*16*16

d4 **=** self**.**Up4**(**d5**)** # 512*32*32

d4 **=** torch**.**cat**((**x4**,**d4**),**dim**=**1**)** # 1024*32*32

d4 **=** self**.**Up_conv4**(**d4**)** # 256*32*32

d3 **=** self**.**Up3**(**d4**)** # 256*64*64

d3 **=** torch**.**cat**((**x3**,**d3**),**dim**=**1**)** # 512*64*64

d3 **=** self**.**Up_conv3**(**d3**)** # 128*64*64

d2 **=** self**.**Up2**(**d3**)** # 128*128*128

d2 **=** torch**.**cat**((**x2**,**d2**),**dim**=**1**)** # 256*128*128

d2 **=** self**.**Up_conv2**(**d2**)** # 64*128*128

d1 **=** self**.**Up1**(**d2**)** # 64*256*256

d1 **=** torch**.**cat**((**x1**,**d1**),**dim**=**1**)** # 128*256*256

d1 **=** self**.**Up_conv1**(**d1**)** # 64*256*256

out **=** self**.**Outconv**(**d1**)**

**return** torch**.**sigmoid**(**out**)**

**Table S1.** Image acquisition protocols of CT scanners involved in the present study.

|  | Peak kilovoltage (kv) | Maximum tube current (mA) | Section thickness (mm) | Reconst-ruction thickness (mm) | Pitch | Contrast agents and amount | Contrast injection rate (mL/s) | Arterial phase range (s) | Porto-venous phase range (s) | Late phase range (s) |
| --- | --- | --- | --- | --- | --- | --- | --- | --- | --- | --- |
| Toshiba Prime  (TPVGH) | 120 | 500 | 0.50 | 5 | 0.81 | Omnipaque 350  90 mL | 2.5 | 40 | 90 | 180 |
| Toshiba Aquilion Prime  (TPVGH) | 120 | 500 | 0.50 | 5 | 0.81 | Omnipaque 350  90 mL | 2.5 | 40 | 90 | 180 |

**Table S2.** The performance of 2D DenseU-Net model for liver segmentation in training, validation and test sets.

| Dataset | Segmentation | |
| --- | --- | --- |
|  | Dice per case (%) | Dice global (%) |
| Training | 97.8 | 97.8 |
| Validation | 95.0 | 95.0 |
| Test | 95.2 | 95.3 |

**Table S3.** Model performances with different phase CT as input

| Phase | Model | Loss |  | Segmentation (dice: %) | | | | | | Detection Rate ( positive / slice number ) | | | | | | False |
| --- | --- | --- | --- | --- | --- | --- | --- | --- | --- | --- | --- | --- | --- | --- | --- | --- |
|  |  |  | pixel | 0~10 | 10~30 | 30~50 | 50~70 | 70~90 | 90~ | 0~10 | 10~30 | 30~50 | 50~70 | 70~90 | 90~ |  |
| A | U-Net | CE |  | 9.77 | 17.38 | 45.92 | 62.14 | 80.95 | 89.19 | 1 / 3 | 67 / 233 | 68 / 108 | 33 / 40 | 18 / 18 | 39 / 39 | 50 / 2170 |
| N | U-Net | CE |  | 8.54 | 8.95 | 30.83 | 63.22 | 67.29 | 86.78 | 1 / 3 | 35 / 233 | 51 / 108 | 34 / 40 | 15 / 18 | 39 / 39 | 32 / 2170 |
| P | U-Net | CE |  | 21.75 | 42.97 | 77.61 | 88.22 | 90.77 | 93.65 | 1 / 3 | 119 / 233 | 95 / 108 | 40 / 40 | 18 / 18 | 39 / 39 | 32 / 2170 |
| Dynamic | U-Net | CE |  | 0 | 39.50 | 72.44 | 75.28 | 80.89 | 91.99 | 0 / 3 | 114 / 233 | 93 / 108 | 36 / 40 | 16 / 18 | 39 / 39 | 28 / 2170 |
| P | DenseU-Net | CE |  | 0 | 43.83 | 74.37 | 81.12 | 92.69 | 93.51 | 0 / 3 | 123 / 233 | 93 / 108 | 39 / 40 | 18 / 18 | 39 / 39 | 35 / 2170 |
| Dynamic | DenseU-Net | CE |  | 0 | 48.49 | 73.55 | 79.34 | 86.13 | 89.05 | 0 / 3 | 142 / 233 | 92 / 108 | 36 / 40 | 17 / 18 | 39 / 39 | 86 / 2170 |

N, non-contrast phase; A, arterial phase; P, portal-venous; Dynamic, dynamic CT images; CE, Cross entropy. Each pixel corresponds to 1.4 mm.

**Table S4.** Model performances with different model architectures

| Phase | Model | Loss |  | Segmentation (dice: %) | | | | | | Detection Rate ( positive / slice number ) | | | | | | False |
| --- | --- | --- | --- | --- | --- | --- | --- | --- | --- | --- | --- | --- | --- | --- | --- | --- |
|  |  |  | pixel | 0~10 | 10~30 | 30~50 | 50~70 | 70~90 | 90~ | 0~10 | 10~30 | 30~50 | 50~70 | 70~90 | 90~ |  |
| Dynamic | U-Net | CE |  | 0 | 39.50 | 72.44 | 75.28 | 80.89 | 91.99 | 0 / 3 | 114 / 233 | 93 / 108 | 36 / 40 | 16 / 18 | 39 / 39 | 28 / 2170 |
| Dynamic | DenseU-Net | CE |  | 0 | 48.49 | 73.55 | 79.34 | 86.13 | 89.05 | 0 / 3 | 142 / 233 | 92 / 108 | 36 / 40 | 17 / 18 | 39 / 39 | 86 / 2170 |
| Dynamic | Hyper-DenseU-Net | CE |  | 0 | 42.43 | 70.62 | 69.80 | 82.32 | 91.42 | 0 / 3 | 122 / 233 | 92 / 108 | 31 / 40 | 16 / 18 | 39 / 39 | 31 / 2170 |
| Dynamic | Single Dense Path U-Net | CE |  | 0 | 33.70 | 61.72 | 70.50 | 84.23 | 89.56 | 0 / 3 | 102 / 233 | 80 / 108 | 34 / 40 | 17 / 18 | 39 / 39 | 49 / 2170 |
| Dynamic | R2U-Net | CE |  | 0 | 14.21 | 41.44 | 74.93 | 71.33 | 74.32 | 0 / 3 | 52 / 233 | 64 / 108 | 36 / 40 | 16 / 18 | 39 / 39 | 172 / 2170 |
| P | U-Net | CE |  | 21.75 | 42.97 | 77.61 | 88.22 | 90.77 | 93.65 | 1 / 3 | 119 / 233 | 95 / 108 | 40 / 40 | 18 / 18 | 39 / 39 | 32 / 2170 |
| P | DenseU-Net | CE |  | 0 | 43.83 | 74.37 | 81.12 | 92.69 | 93.51 | 0 / 3 | 123 / 233 | 93 / 108 | 39 / 40 | 18 / 18 | 39 / 39 | 35 / 2170 |
| P | R2U-Net | CE |  | 23.49 | 39.93 | 64.42 | 70.21 | 77.81 | 70.84 | 1 / 3 | 116 / 233 | 82 / 108 | 39 / 40 | 17/ 18 | 38 / 39 | 111 / 2170 |

N, non-contrast phase; A, arterial phase; P, portal-venous; Dynamic, dynamic CT images; CE, Cross entropy. Each pixel corresponds to 1.4 mm.

**Table S5.** Model performances with different loss functions

| Phase | Model | Loss |  | Segmentation (dice: %) | | | | | | Detection Rate ( positive / slice number ) | | | | | | False |
| --- | --- | --- | --- | --- | --- | --- | --- | --- | --- | --- | --- | --- | --- | --- | --- | --- |
|  |  |  | pixel | 0~10 | 10~30 | 30~50 | 50~70 | 70~90 | 90~ | 0~10 | 10~30 | 30~50 | 50~70 | 70~90 | 90~ |  |
| Dynamic | DenseU-Net | CE |  | 0 | 48.49 | 73.55 | 79.34 | 86.13 | 89.05 | 0 / 3 | 142 / 233 | 92 / 108 | 36 / 40 | 17 / 18 | 39 / 39 | 86 / 2170 |
| Dynamic | DenseU-Net | FL |  | 0 | 49.26 | 74.31 | 71.41 | 83.34 | 93.02 | 0 / 3 | 143 / 233 | 93 / 108 | 32 / 40 | 18 / 18 | 39 / 39 | 63 / 2170 |
| Dynamic | DenseU-Net | MFB |  | 34.02 | 41.43 | 70.99 | 77.61 | 83.94 | 91.58 | 2 / 3 | 181 / 233 | 106 / 108 | 40 / 40 | 18 / 18 | 39 / 39 | 693 / 2170 |
| Dynamic | DenseU-Net | FL + DL |  | 0 | 51.51 | 74.39 | 74.33 | 88.50 | 92.90 | 0 / 3 | 147 / 233 | 94 / 108 | 35 / 40 | 18 / 18 | 39 / 39 | 42 / 2170 |
| Dynamic | DenseU-Net | MBF + DL |  | 0 | 45.25 | 77.93 | 79.68 | 84.97 | 94.04 | 0 / 3 | 150 / 233 | 103 / 108 | 39 / 40 | 17 / 18 | 39 / 39 | 278 / 2170 |
| P | U-Net | CE |  | 21.75 | 42.97 | 77.61 | 88.22 | 90.77 | 93.65 | 1 / 3 | 119 / 233 | 95 / 108 | 40 / 40 | 18 / 18 | 39 / 39 | 32 / 2170 |
| P | U-Net | CE + DL |  | 23.29 | 42.58 | 75.19 | 77.30 | 89.25 | 93.19 | 1 / 3 | 120 / 233 | 93 / 108 | 35 / 40 | 18 / 18 | 39 / 39 | 33 / 2170 |

N, non-contrast phase; A, arterial phase; P, portal-venous; Dynamic, dynamic CT images; CE, cross entropy; FL, focal loss; MFB, median frequency balance; DL, dice loss. Each pixel corresponds to 1.4 mm.

**Table S6.** Model performances compared with 2D HFS-Net and 3D HFS-Net

| Phase | Model | Loss |  | Segmentation (dice: %) | | | | | | Detection Rate ( positive / slice number ) | | | | | | False |  |
| --- | --- | --- | --- | --- | --- | --- | --- | --- | --- | --- | --- | --- | --- | --- | --- | --- | --- |
|  |  |  | pixel | 0~10 | 10~30 | 30~50 | 50~70 | 70~90 | 90~ | 0~10 | 10~30 | 30~50 | 50~70 | 70~90 | 90~ |  | |
| P | U-Net | CE |  | 21.75 | 42.97 | 77.61 | 88.22 | 90.77 | 93.65 | 1 / 3 | 119 / 233 | 95 / 108 | 40 / 40 | 18 / 18 | 39 / 39 | 32 / 2170 | |
| Dynamic | DenseU-Net | FL + DL |  | 0 | 51.51 | 74.39 | 74.33 | 88.50 | 92.90 | 0 / 3 | 147 / 233 | 94 / 108 | 35 / 40 | 18 / 18 | 39 / 39 | 42 / 2170 | |
| Dynamic | 2D HFS-Net | FL + DL |  | 0 | 55.65 | 78.34 | 76.65 | 91.20 | 92.68 | 0 / 3 | 162 / 233 | 99 / 108 | 36 / 40 | 18 / 18 | 39 / 39 | 126 / 2170 | |
| Dynamic | 3D HFS-Net | FL + DL |  | 18.10 | 56.53 | 81.57 | 79.67 | 91.21 | 93.49 | 1 / 3 | 168 / 233 | 104 / 108 | 37 / 40 | 18 / 18 | 39 / 39 | 76 / 2170 | |

FL, focal loss; DL, dice loss; 2D HFS-Net, HFS-Net contains hierarchical but not fusion strategy; 3D HFS-Net, the complete architecture of HFS-Net. Each pixel corresponds to 1.4 mm.

**Table S7.** Performance of the HFS-Net model for HCC detection and segmentation in the training, validation and test sets

| Dataset | Segmentation | | | | Detection Sensitivity | | | |
| --- | --- | --- | --- | --- | --- | --- | --- | --- |
|  | Dice per case (%) | Dice global (%) | MTD MAE (cm) | Per tumor volume (%) | | Per tumor cut (%) | Per slice (%) | Per case (%) |
| Training | 77.6 | 88.8 | 0.41 | 97.1 | | 96.4 | 96.8 | 99.0 |
| Validation | 59.2 | 79.0 | 1.52 | 94.1 | | 82.5 | 82.7 | 93.2 |
| Test | 58.7 | 82.8 | 0.73 | 91.4 | | 82.1 | 84.3 | 92.2 |

**REFERENCES**

1. Kayalibay, B.; Jensen, G.; van der Smagt, P., CNN-based segmentation of medical imaging data. *arXiv preprint arXiv:1701.03056* **2017**.

2. Ronneberger, O.; Fischer, P.; Brox, T. In *U-net: Convolutional networks for biomedical image segmentation*, International Conference on Medical image computing and computer-assisted intervention, Springer: 2015; pp 234-241.

3. Huang, G.; Liu, Z.; Van Der Maaten, L.; Weinberger, K. Q. In *Densely connected convolutional networks*, Proceedings of the IEEE conference on computer vision and pattern recognition, 2017; pp 4700-4708.

4. Dong, R.; Pan, X.; Li, F., DenseU-net-based semantic segmentation of small objects in urban remote sensing images. *IEEE Access* **2019,** *7*, 65347-65356.

5. De Boer, P.-T.; Kroese, D. P.; Mannor, S.; Rubinstein, R. Y., A tutorial on the cross-entropy method. *Annals of operations research* **2005,** *134* (1), 19-67.

6. Lin, T.-Y.; Goyal, P.; Girshick, R.; He, K.; Dollár, P. In *Focal loss for dense object detection*, Proceedings of the IEEE international conference on computer vision, 2017; pp 2980-2988.

7. Milletari, F.; Navab, N.; Ahmadi, S.-A. In *V-net: Fully convolutional neural networks for volumetric medical image segmentation*, 2016 fourth international conference on 3D vision (3DV), IEEE: 2016; pp 565-571.

8. Zhu, W.; Huang, Y.; Zeng, L.; Chen, X.; Liu, Y.; Qian, Z.; Du, N.; Fan, W.; Xie, X., AnatomyNet: deep learning for fast and fully automated whole‐volume segmentation of head and neck anatomy. *Medical physics* **2019,** *46* (2), 576-589.
